# Effects of Different Levels of Licorice Residue and Sweet Sorghum on Pellet Feed Quality, Intestinal Morphology, Cecal Volatile Fatty Acids, and Microorganisms in Meat Rabbits

**DOI:** 10.3390/microorganisms14040868

**Published:** 2026-04-12

**Authors:** Jie Wan, Mingxin Zhao, Qihui Wu, Xin Ren, Lianqun Wang, Chongpeng Bi, Sujiang Zhang

**Affiliations:** 1 Key Laboratory of Livestock and Grass Resources Utilization Around Tarim, Ministry of Agriculture and Rural Areas (Co-Construction by Ministries and Provinces), Key Laboratory of Tarim Animal Husbandry Science and Technology, College of Animal Science and Technology, Tarim University, Alar 843300, China; 17793379711@163.com (J.W.);; 2 College of Animal Science and Technology, Northeast Agricultural University, Harbin 150030, China

**Keywords:** licorice residue, sweet sorghum, pellet feed quality, intestinal morphology, cecal microorganisms

## Abstract

This study evaluated how varying proportions of licorice residue and sweet sorghum affect pellet quality, growth performance, intestinal morphology, and cecal microflora in 120 healthy 30-day-old Ira rabbits, which were randomly assigned to five groups (six replicates of four rabbits each). Five experimental diets were formulated, each containing 30% licorice residue and sweet sorghum (with licorice residue at 0%, 25%, 50%, 75%, or 100% *w*/*w*) and 70% other components. We found that licorice residue level significantly affected pellet hardness, powder content, and volumetric weight (*p* < 0.05). The L25 group had significantly higher final body weight (FBW) and average daily gain (ADG) than other groups (*p* < 0.05). In the duodenum, villus height (VH) was improved in L25 (*p* < 0.05). Ileal VH increased significantly in L0, L25 and L50 (*p* < 0.05). At the phylum level, Firmicutes were most enriched. At the genus level, Faecousia and SFMI01 abundance increased with higher licorice residue. LEfSe analysis confirmed that varying licorice residue levels influenced cecal microbial composition from phylum to genus. The addition of 25% licorice residue to the diet can improve the growth performance of meat rabbits and improved both intestinal tissue morphology and the cecal microbiota of meat rabbits.

## 1. Introduction

On a global scale, rabbit farming is regarded as one of the most efficient and sustainable major development industries; it has the advantages of rapid growth and fast reproduction and can also meet the market demand for meat [1]. Over recent years, through technological advances and large-scale farming, the rabbit industry has transitioned from small-scale to large-scale breeding, with a steady increase in rabbit meat production. China is one of the most important rabbit meat producing countries in the world [2]. Along with Italy, Spain, France and other countries, China ranks among the top countries in global rabbit meat production [3]. Its rabbit farming industry occupies an important position in agricultural production. In this context, the Chinese rabbit farming industry for meat consumption has formed large-scale farming settlements in Fujian, Sichuan and Xinjiang and is becoming an emerging sector for local rural revitalization [4,5]. However, in the arid desert area of southern Xinjiang, the shortage of roughage has adversely affected the development of the animal husbandry industry. The use of local feed resources could serve as an alternative to reduce the occurrence of this problem. As a result, it is essential to develop unconventional roughage sources that utilize local feed resources for supporting animal husbandry in this region.

Sweet sorghum, an annual herbaceous plant belonging to the family Gramineae, has strong regenerative properties and can thus be harvested extensively [6]. It is an important silage crop with cold and drought resistance, salt and alkali resistance and stress tolerance [7]. In terms of feeding value, it has the advantages of high nutritional value, since it is abundant in protein and various minerals, good palatability and high yield [8]. The straw that remains post-harvest is rich in amino acids (AAs), vitamins, minerals, and soluble sugars [9], making it a high-quality forage.

Licorice, a perennial herb that grows in arid and semi-arid climates, possesses immunomodulatory, antibacterial, antioxidant, anti-inflammatory, antiviral, and anti-infective properties [10]. Licorice is rich in active ingredients such as glycyrrhizic acid, flavonoids and polysaccharides [11] and is mainly used in the fields of medicine and food. Licorice residue, the material remaining after extraction of glycyrrhizic acid, flavonoids, and other active compounds, contains cellulose, hemicellulose, lignin [12], and residual bioactive components, including proteins, flavonoids, triterpenes, and AAs. Previous studies, such as Camila et al. [13], have shown that licorice bioactives can exert therapeutic effects on intestinal disorders. However, its use is restricted by its high content of crude fiber and poor palatability. Therefore, combining sweet sorghum and licorice residue into pellet feed may enhance the nutritional value of the feed while supporting animal growth and intestinal health. However, these resources remain underutilized in rabbit farming. Thus, in this study, the effects of different proportions of licorice residue and sweet sorghum in diets on the intestinal morphology, pH, and cecal microbiota of meat rabbits were examined, providing a scientific basis for the rational use of these local feed resources.

## 2. Materials and Methods

### 2.1. Test Materials

The licorice residue used in this study was obtained from the New Agricultural Licorice Factory in Alar, Xinjiang (longitude 81.285884, latitude 40.541914). Sweet sorghum (variety: Cowley; Sugar brix: 25.2%) was grown at the experimental station of Tarim University; after harvesting, fresh straw was uniformly dried and ground into forage powder. The nutrient compositions of the licorice residue and sweet sorghum are presented in Table 1.

### 2.2. Experimental Diets

According to the nutritional level recommended in ‘Nutrition of the rabbit’ [14] and the characteristics of local feed resources, the experimental diet formula of each group was designed. The pellet feed was produced using a 9KJ-350 pellet feed press (Hexie Feed Machinery Manufacturing Co., Ltd., Xinxiang, China), resulting in pellets with a diameter of 3 mm and a length of 20–23 mm. The composition and analysis of the basal diet are presented in Table 2.

### 2.3. Chemical Analysis of Diet

Dry matter (DM) content was determined using the oven-drying method (AOAC, 934.01). The ether extract (EE) content was determined by Soxhlet extraction (AOAC, 989.05). Ash content was determined by high-temperature incineration (AOAC, 935.42). The Kjeldahl method was employed to measure the crude protein (CP) content. The contents of neutral-detergent fiber (NDF) and acid-detergent fiber (ADF) were determined by the method of Van Soest et al. [15]. The concentrations of calcium (Ca) were determined by atomic absorption spectrophotometry, and phosphorus (P) was determined by colorimetry [16]. Digestible energy was calculated according to ‘Nutrition of the rabbit’.

### 2.4. Experimental Design and Feeding Management

A total of 120 healthy Ira meat rabbits (30-day-old) with similar body weights (equal numbers of males and females) were randomly assigned to five groups, each with six replicates and four rabbits per replicate. The ratio of licorice residue to sweet sorghum in each group was 0:100 (L0), 25:75 (L25), 50:50 (L50), 75:25 (L75), and 100:0 (L100), respectively, and these were prepared into pellet feed. The experiment consisted of a 7-day acclimatization period, followed by a 42-day feeding trial.

This study was conducted at the Experimental Station of the College of Animal Science and Technology, Tarim University. The experimental protocol used in this study (No: PB20251226003) has been approved by the Animal Experimental Ethics Committee of Tarim University. Before the trial, the rabbit house and cages were thoroughly disinfected. The size of each rabbit cage was 42 cm × 50 cm × 35 cm, with the rabbits housed individually, and indoor temperatures were maintained at 20–24 °C. All rabbits received routine immunization before the experiment. They were fed twice daily at 10:00 and 20:00, had ad libitum access to drinking water, and were maintained under natural lighting and ventilation. The rabbit house was cleaned regularly throughout the trial.

### 2.5. Determination Index and Methods

#### 2.5.1. Processing Quality Indicators of Pellet

After pellet preparation, representative samples from each dietary group were collected to evaluate pellet quality using the following indicators:

(1) Briquetting ratio

Based on GB/T 16765-1997, 500 g of pellets was collected at the granulator outlet, dried, and screened using a 0.8× sieve (2.5 mm). The briquetting ratio was calculated as the percentage of material retained on the sieve relative to the total sample weight.

(2) Measurement of length

Twenty uniformly sized pellets were randomly selected from each group. After smoothing both ends with sandpaper, pellet length was measured using a vernier caliper (Ningbo Deli Tools Co., Ltd., Ningbo, China).

(3) Pellet hardness

Twenty pellets per group were randomly selected and compressed until broken using a GWJ-III grain hardness meter (Jiangxi Maitu Instrument Co., Ltd., Nanchang, China). The mean value was recorded as pellet hardness.

(4) Powder content

Feed samples (1.5 kg) were taken and placed in a 10-mesh sieve (pore size 1.4 mm), according to Zhang (2016) [17]. The sieve was manually screened and weighed. The powder content was the percentage of sieve weight to the total sample weight.

(5) Pulverization rate

Based on GB/T 16765-1997, two 500 g portions of the sieved material from the powder-content test were placed into a two-chamber pulverizer and rotated at 50 rpm for 10 min. The material was then rescreened using a 10-mesh sieve, and the pulverization rate was calculated as the percentage of material passing through the sieve relative to the total sample weight.

(6) Volumetric weight

Pellets were poured into a 1000 mL graduated cylinder until reaching the 1000 mL mark, after which the sample was weighed. Volume-weight was calculated as the ratio of pellet mass to volume.

#### 2.5.2. Growth Performance Test

The rabbits were fasted on the day before the 0 day and 42 day of the formal experiment. The fasting body weight of the meat rabbits was measured, and the initial body weight (IBW), final body weight (FBW), and average daily gain (ADG) were calculated. During the experiment, the daily feed intake was accurately recorded, and the average daily feed intake (ADFI) was calculated. According to the ADFI and ADG, the ratio of material to weight (F/G) was calculated. The calculation formula is as follows:

ADG (g/d) = (FBW − IBW)/Day

ADFI (g/d) = Total Feed Intake (TFI)/Day

F/G = ADFI/ADG

#### 2.5.3. Intestinal Tissue Morphology

At the end of the experiment, all rabbits were fasted for 12 h. Two rabbits (one male and one female) with similar body weight were randomly selected from each replicate and slaughtered according to Blasco et al. [18]. The duodenum, jejunum, and ileum were quickly excised, the contents were removed, and each segment was rinsed with normal saline. A 2–3 cm section from each intestinal segment was fixed in 4% paraformaldehyde and processed for routine histological examination, including the steps of trimming, dehydration, clearing, embedding, sectioning, dewaxing, hydration, staining, dehydration, and mounting. Intestinal morphology was microscopically (40×) examined, and images of five representative fields were acquired. Villus height (VH), villus width (VW), crypt depth (CD), muscle thickness (MT), and the V/C ratio were measured using the Image-Pro plus 6.0 (Media Cybernetics, Inc., Rockville, MD, USA) analysis system.

#### 2.5.4. Determination of Cecal Volatile Fatty Acids (VFAs)

Cecal pH was measured three times using a pH meter (Shanghai LeiMag PHSJ-4A, Shanghai, China), and the mean value was recorded. For analyzing VFAs, 0.5 g of cecal content was diluted threefold with distilled water and centrifuged at 5400 rpm for 10 min at 4 °C. The supernatant (1 mL) was mixed with 0.2 mL of 25% metaphosphoric acid containing the internal standard 2-ethyl butyric acid (2EB), placed on ice for ≥30 min and centrifuged again under the same conditions to remove protein precipitates. The supernatant was analyzed using a Trace 1310 gas chromatograph (Thermo Fisher Scientific, Waltham, MA, USA) to determine VFA. The chromatograph was fitted with a capillary column Agilent HP-INNOWAX (30 m × 0.25 mm ID × 0.25 μm) (Santa Clara, CA, USA), and helium was used as the carrier gas at 1 mL/min. Injection was made in split mode at 10:1, with an injection volume of 1 μL and an injector temperature of 250 °C. The temperatures of the ion source and MS transfer line were 300 °C and 250 °C, respectively. The column temperature was programmed to increase from an initial temperature of 90 °C to 120 °C at 10 °C/min, then to 150 °C at 5 °C/min, and, finally, to 250 °C at 25 °C/min, which was maintained for 2 min.

#### 2.5.5. Determination of Cecal Microorganisms

Cecal contents were collected into sterile 5 mL cryotubes, immediately frozen in liquid nitrogen, and stored at −80 °C. The samples were transported on dry ice to Nanjing Parsons Gene Technology Co, Ltd., China for DNA extraction and amplification of target regions by PCR. Sequencing libraries were then prepared using the Illumina TruSeq Nano DNA LT Library Prep Kit (Illumina, San Diego, CA, USA), followed by high-throughput sequencing. Raw reads were processed to remove primer sequences, low-quality reads were filtered and denoised, paired-end reads were merged, and chimeras were eliminated. Taxonomic classification of amplicon sequence variants was performed using the QIIME2 feature-classifier plugin (Greengenes database trimmed to the V3–V4 region; primers, 338F/806R). Microbial alpha diversity, beta diversity, and relative abundance were analyzed using QIIME2 (2019.4) and Mothur1.48.3 software.

### 2.6. Data Processing and Statistical Analysis

Data were analyzed using SPSS 19.0. Data are shown as mean and standard error of the mean (SEM). Variable distributions were evaluated using the Shapiro–Wilk test. Normally distributed variables were compared using Duncan’s method test, while non-normally distributed variables were analyzed using the Kruskal–Wallis H test for overall comparisons. When the overall test was significant, post hoc pairwise comparisons were performed using Dunn’s test with Bonferroni correction. Linear and quadratic analyses were conducted to evaluate trends in pellet quality, intestinal morphology, and cecal VFAs. *p*-values < 0.05 were considered statistically significant.

## 3. Results

### 3.1. Pellet Feed Quality

As shown in Table 3, pellet length and briquetting ratio did not differ significantly among the five groups (*p* > 0.05). Pellet hardness in the L100 groups was significantly higher than in the other groups (*p* < 0.05), exhibiting a quadratic trend (initial decrease until L75, followed by an increase in L100) with increasing licorice residue inclusion. Powder content in the L50 and L75 groups was significantly higher than in the remaining three groups (*p* < 0.05). The pulverization rate of the L50 and L25 groups was numerically higher than that of the L0, L75, and L100 groups, although the differences were not significant (*p* > 0.05). The volume-weight of pellet feed in the L0 group was significantly higher than in the other groups (*p* < 0.05), while the L25 group was the lowest (561.14 g). The powder content and volume-weight of the five groups of pellets showed a quadratic curve trend of decreasing first and then increasing with the increase in licorice residue ratio.

### 3.2. Growth Performance

As shown in Table 4, there was no significant difference in initial body weight (IBW) and average daily feed intake (ADFI) (*p* > 0.05), but the FBW and ADG of the L25 group were significantly higher than those of the other groups (*p* < 0.05), and the ADG increased first and then decreased with the increase in licorice residue ratio. At the same time, the F/G of the L25 group was significantly the lowest (*p* < 0.05).

### 3.3. Intestinal Morphology of Meat Rabbits

Table 5 shows that, in the duodenum, VW and CD did not differ significantly among groups (*p* > 0.05), whereas VH in the L25 group was significantly higher than in the other groups (*p* < 0.05). The V/C ratios of the L0, L25, and L75 groups were significantly higher than those of the L50 and L100 groups (*p* < 0.05). Muscle thickness (MT) was significantly higher in the L75 group and exhibited a quadratic trend of decreasing and then increasing with rising proportions of licorice residue (*p* < 0.05). In the jejunum, VH and V/C ratios showed no significant differences among groups (*p* > 0.05), while CD in the L25 group was significantly higher than in the remaining groups (*p* < 0.05). MT in the L25 and L75 groups was significantly greater than in the other groups (*p* < 0.05), and VW was significantly higher in the L50 group (*p* < 0.05). Both VW and MT increased initially and then decreased in a quadratic pattern as the proportion of licorice residue increased (*p* < 0.05). In the ileum, CD and MT did not differ significantly across treatments (*p* > 0.05), while VH in the L0 and L25 groups was significantly higher than in the L75 and L100 groups and showed a linear decrease with increasing licorice residue (*p* < 0.05). VW and V/C ratios were significantly higher in the L50 and L100 groups (*p* < 0.05), and VW increased linearly with increasing licorice residue proportion (*p* < 0.05).

### 3.4. Cecal Volatile Fatty Acids in Meat Rabbits

As presented in Table 6, no significant differences were observed in pH or NH_3_-N levels among treatments (*p* > 0.05), with cecal pH ranging from 6.85 to 7.08. The AA content of the L50 group was significantly higher than that of all other groups (*p* < 0.05), and AA levels in the L100 group were significantly lowest (*p* < 0.05). AA content exhibited a quadratic trend, increasing initially and then decreasing with higher proportions of licorice residue (*p* < 0.05). No significant differences were detected among groups for BA, PA, VA, IVA, IBA, or HA (*p* > 0.05).

### 3.5. Cecal Microbial Diversity of Meat Rabbits

#### 3.5.1. Alpha Diversity Analysis of Cecal Microflora

**Figure 1:**
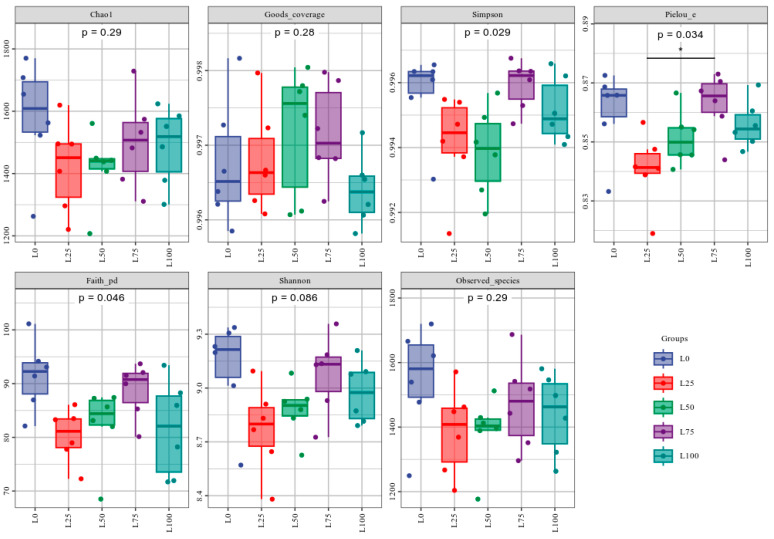
Alpha diversity of cecal microorganisms in meat rabbits treated with different proportions of licorice residue and sweet sorghum. Each panel in the gray area corresponds to an alpha diversity index. The abscissa is the grouping label, and the ordinate is the value of the corresponding alpha diversity index. Each color in the boxplot represents each group, each point in the figure represents a sample, and the same group of samples is represented by the same color. n = 6. * *p* < 0.05.

The α-diversity indices of cecal bacterial communities, based on 16S rRNA gene sequencing, are shown in Figure 1 and Supplementary Table S1. The Goods coverage values exceeded 0.99 for all samples, indicating that the sequencing depth was sufficient to capture the overall microbial diversity. The Faith’s PD index of the L0 group was significantly higher than that of the L25, L50 and L100 groups (*p* < 0.05), while the Pielou’s evenness index of the L0, L75 and L100 groups was significantly higher than that of the L25 group, and the Simpson index was significantly higher than that of the L25 and L50 groups (*p* < 0.05). Meanwhile, no significant differences were observed among treatments for the Chao1, Goods-coverage, observed-species, or Shannon indices (*p* > 0.05).

#### 3.5.2. Analysis of Bacterial Diversity of Cecal Microbial Flora

**Figure 2:**
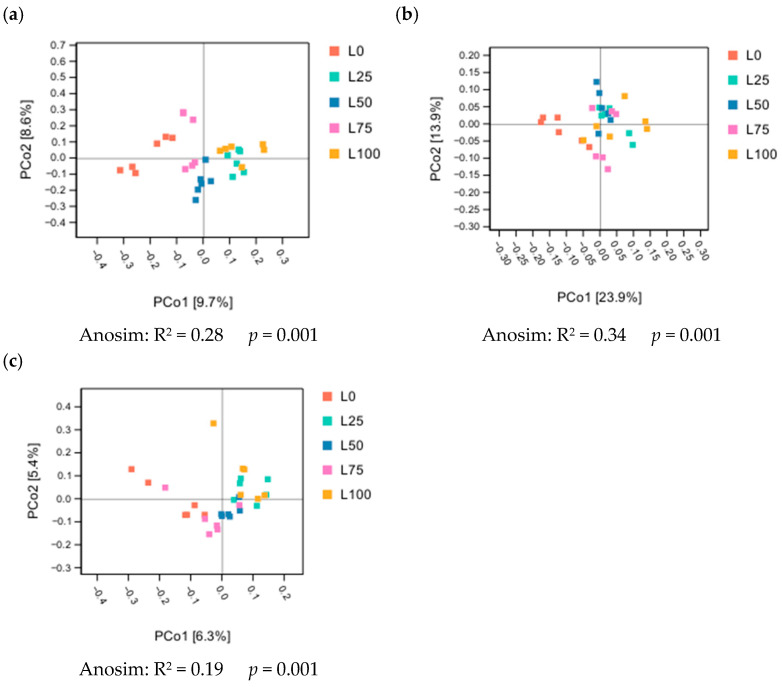
Analysis of the beta diversity of cecal microbes in meat rabbits treated with different proportions of licorice residue and sweet sorghum. Based on bary _ curtis (**a**), unweighted _ unifrac distance (**b**), and weighted _ unifrac distance (**c**). Each point in the figure represents a sample, and samples belonging to the same group are represented by the same color. n = 6.

Principal coordinate analysis (PCoA) revealed clear separation of cecal microbial communities among rabbits fed different proportions of licorice residue and sweet sorghum straw. This pattern was consistently supported by three β-diversity metrics, Bray–Curtis (Figure 2a), weighted UniFrac (Figure 2b), and unweighted UniFrac (Figure 2c), confirming distinct microbial community structures across dietary treatments.

#### 3.5.3. Analysis of Operational Taxonomic Units (OTUs)

**Figure 3:**
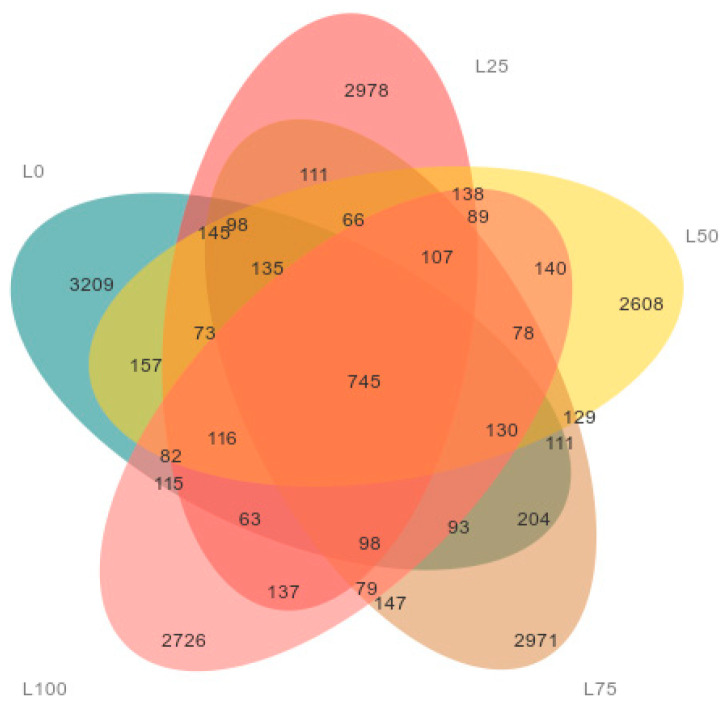
Operational taxonomic units analysis of cecal microflora in rabbits with different proportions of licorice residue and sweet sorghum (Venn diagram). Each color block represents a group, and the overlapping area between the color blocks indicates the OTUs shared by the corresponding groups. The number of OTUs in each block is indicated. n = 6.

Common and unique OTUs across the five treatment groups were analyzed, and the corresponding Venn diagram was constructed (Figure 3). The total number of OTUs identified in the L0, L25, L50, L75, and L100 groups was 5574, 5244, 5036, 5447, and 4843, respectively. The number of unique OTUs was 3209, 2978, 2608, 2971 and 2726 for the L0, L25, L50, L75 and L100 groups, respectively, indicating substantial diet-dependent variation in microbial composition.

#### 3.5.4. Distribution of Cecal Bacterial Community at Phylum and Genus Levels

**Figure 4:**
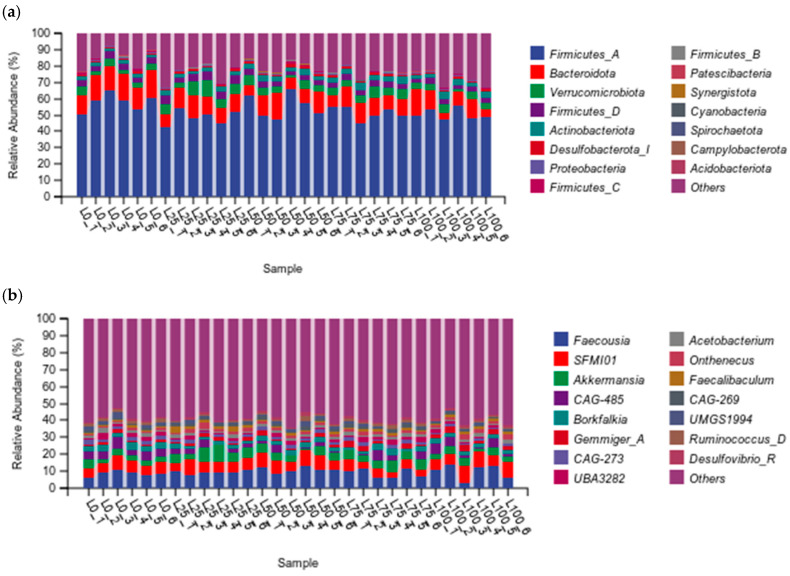
Effects of different proportions of licorice residue and sweet sorghum on the composition of the cecal microflora in meat rabbits. Relative abundance at the phylum (**a**) and genus (**b**) levels. The abscissa in the figure is the sample and name of the grouping scheme, and the ordinate is the relative abundance of each classification unit at a specific classification level. n = 6.

Figure 4 illustrates the distribution of the cecal bacterial community in meat rabbits. At the phylum level (Figure 4a and Table S2 (Supplementary Materials)), Firmicutes_A accounted for the highest proportion, reaching 48.74–57.90%, followed by Bacteroidota (9.68–14.72%) and Verrucomicrobiota (3.55–6.60%). Among these, the relative abundance of Firmicutes_A in the L0 group was significantly higher than that in the L25, L75 and L100 groups (*p* < 0.05). Although the L0 treatment group also showed the highest relative abundance of Bacteroidota, the difference was not significant (*p* > 0.05). With the increase in licorice residue proportion, the relative abundance of Verrucomicrobiota and Firmicutes_D in the L25 treatment group reached the highest value, followed by a decline, while the relative abundance of Actinobacteriota exhibited a linear increase with increasing levels of licorice residue until L75.

At the genus level (Figure 4b and Table S3), Faecousia was the predominant genus across treatments (8.67–11.13%), with the L50 group showing the highest abundance, although differences were not significant (*p* > 0.05). The relative abundances of Akkermansia and Faecalibaculum were significantly higher in the L25 group compared with their abundances in the L0, L50, and L100 groups (*p* < 0.05). Meanwhile, Gemmiger_A increased progressively with increasing licorice residue proportion, reaching its highest abundance of 3.01% in the L100 group.

#### 3.5.5. Linear Discriminant Analysis Effect Size (LEfSe) Analysis

**Figure 5:**
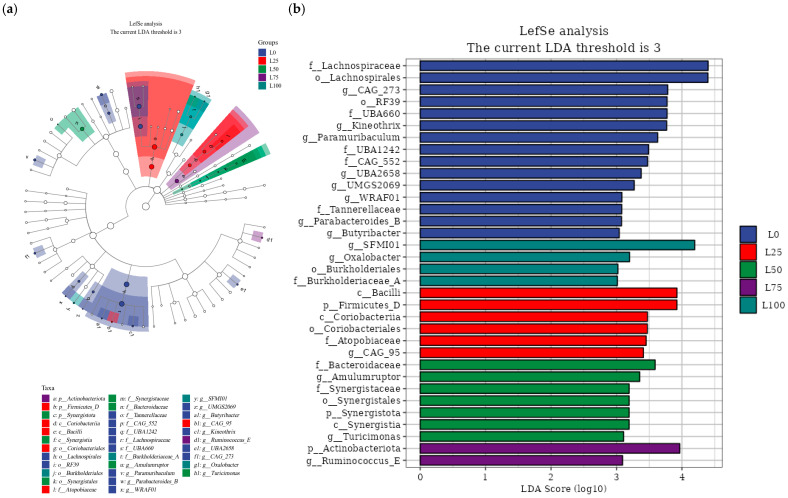
The linear judgment analysis combined with the effect size (LEfSe) was used to determine the classification unit having the largest differential abundance in meat rabbits’ cecal microflora with different proportions of licorice residue and sweet sorghum pellet feed. (**a**) LEfSe analysis of 16S rRNA sequencing yielded a taxonomic branch diagram showing the hierarchy of the main taxa from phylum to genus (from the inner to outer ring). The size of each node corresponds to the average relative abundance of the taxon. Hollow nodes indicate that the differences between groups are not significant, whereas the nodes of other colors represent taxa reflecting significant between-group differences. Nodes with the same color represent the same treatment group, and the letters identify the taxa exhibiting significant differences. (**b**) Only those taxa whose linear discriminant analysis (LDA) score reached the significant threshold of 3 are displayed. n = 6.

A combination of LEfSe analysis (Figure 5a) and linear discriminant analysis (LDA) (Figure 5b) was used to assess differences in microbial abundance in the overall community at both the phylum and genus levels; the LDA threshold used was ≥3. The results showed the presence of 15 discriminatory taxa in the L0 group, including Lachnospirales and RF39 at the order level; Lachnospiraceae, UBA660, CAG552, UBA1242, and Tannerellaceae at the family level; and Kineothrix, CAG273, Paramuribaculum, UBA2658, UMGS2069, Parabacteroides, WRAF01, and Butyribacter at the genus level. In the L25 group, significantly enriched taxa were Firmicutes, Bacilli, Coriobacteriales, Coriobacteriia, Atopobiaceae, and CAG-95. The L50 group showed enrichment of Synergistota, Synergistia, Synergistales, Synergistaceae, Bacteroidaceae, Amulumruptor, and Turicimonas. In the L75 group, only two discriminatory taxa, Actinobacteriota and Ruminococcus, were identified. In the L100 group, Burkholderiales, Burkholderiaceae, SFMI01, and Oxalobacter were more abundant.

## 4. Discussion

### 4.1. Pellet Feed Quality for Meat Rabbits

High quality pellet feed reduces feed waste, improves feed utilization, and is also closely linked to the growth performance and overall health of meat rabbits [19]. Its processing quality is generally evaluated using indicators such as pellet length, hardness, briquetting ratio, powder content, pulverization rate, and volume-weight. Among these, pellet length and hardness are key factors affecting palatability and digestibility, while the ratio of briquetting reflects pellet integrity and stability. Pellets with a higher briquetting ratio are more durable and less prone to breakage, thereby minimizing feed loss. Although pellet length does not significantly affect the growth performance of meat rabbits, an appropriate pellet length can reduce feed waste, likely due to improved pellet stability [20]. Prayoga et al. [21] found that replacing soybean meal with 15% leucaena leaf powder significantly increased pellet hardness, while another study showed no significant difference in briquetting ratio when legumes, such as Moringa oleifera and leucaena, were used as protein sources [22]. In the present experiment, pellet length and briquetting ratio did not differ significantly among the groups, and the results are basically consistent with the above test results [21], whereas feed hardness in the L100 group was significantly higher than in the other treatments. The reason for this result is that, on the one hand, licorice residue has a high lignin content. In the production process, high temperature and high pressure will soften the lignin, forming a natural adhesive, and the lignin will re-solidify after cooling, so that the pellet feed reaches a hard degree. On the other hand, the structure of cellulose and hemicellulose in licorice residue is relatively short, and the density is high. After crushing, the short fiber is more easily combined with lignin during the granulation process, thus forming a high-hardness pellet feed. Powder content and pulverization rate are important indicators of pellet quality, as excessive powder not only reduces palatability and nutritional value but may also cause respiratory issues in livestock [23] and a high pulverization rate can increase feed loss and affect feed intake. In this study, the pulverization rate of all five treatments ranged from 1.18% to 3.53%, meeting the GB/T 16765-1997 standard (≤10%), with no significant differences among groups; however, differences in powder content were significant. The results also showed that the volume-weight decreased markedly after the inclusion of licorice residue but increased gradually with higher proportions of licorice residue. This may be attributed to the material density of licorice residue, which could reduce internal particle gaps and improve overall feed volume-weight, indicating that licorice residue can significantly alter pellet density.

### 4.2. Growth Performance of Meat Rabbits

Growth performance is the core index to measure the efficiency of animal production, and it is also an intuitive manifestation of animal growth and nutrient absorption during feeding, mainly including average daily gain and average daily feed intake. F/G is the ratio of feed consumption to animal weight gain, and its level directly reflects the effectiveness of nutrient utilization, that is, lower F/G means higher feed utilization and lower feeding cost [24]. In this study, when the addition of licorice residue was 25%, the ADG of Ira meat rabbits increased, and the F/G decreased. This result may be explained by the higher cellulose content of licorice residue, which stimulates intestinal peristalsis and helps maintain normal gastrointestinal function [25]. Meanwhile, the abundance of vitamins and soluble sugars in sweet sorghum enhances feed palatability for animals [7]. When a balance between the two components is established, animal feed intake may be maintained or even enhanced. Furthermore, licorice residue contains certain bioactive substances that can effectively prevent digestive tract diseases and facilitate the growth and development of animals [26].

### 4.3. Intestinal Morphology of Meat Rabbits

The intestine is the primary site for nutrient digestion and absorption in animals and an essential component of the immune system in meat rabbits. A healthy intestinal tract not only enhances nutrient absorption but also supports immune function [27]. VW helps maintain tight epithelial junctions, thereby reducing pathogen invasion [28], whereas CD reflects the renewal rate of intestinal epithelial cells; a lower CD indicates faster cell turnover, leading to a higher villus height-to-crypt depth (V/C) ratio and stronger digestive and absorptive capacity [29]. MT is typically related to intestinal motility and barrier function. A thicker muscle layer is associated with greater intestinal motility [30]. In this study, it was found that the VH, VW, CD, MT and V/C values altered with the addition of different proportions of licorice residue. These results align with the findings reported by You et al. [31], who reported that dietary licorice flavonoid powder increased VH, reduced CD, and increased the V/C ratio, thereby enhancing intestinal absorption. Similarly, Qiao et al. [32] found that supplementing broiler diets with 150 mg/kg licorice polysaccharide increased VH and VW, decreased CD, and improved the V/C ratio. This result may be attributed to the fact that the polysaccharides and saponins in licorice possess antioxidant and immunomodulatory properties, which can reduce oxidative stress in the intestine, thereby protecting intestinal epithelial cells against damage [33] and enhancing nutrient absorption and metabolism [32]. Therefore, the combination of licorice residue and sweet sorghum straw has a certain improvement effect on the intestinal tissues of meat rabbits.

### 4.4. Cecal Volatile Fatty Acids in Meat Rabbits

VFAs are the primary metabolites produced by cecal microbial fermentation of dietary fiber [34]. These metabolites promote intestinal epithelial cell proliferation and repair, thereby improving gut health [35], and also inhibit the growth of harmful bacteria by modulating intestinal pH, contributing to the maintenance of microbial homeostasis [36]. Additionally, VFA production reduces cecal pH and lowers ammonia nitrogen (NH_3_-N) accumulation, thereby alleviating the inhibitory effects of ammonia on microbial activity [37]. In the present study, different ratios of licorice residue and sweet sorghum had no significant impact on cecal pH or NH_3_-N concentrations, but they notably influenced AA profiles. When the proportion of licorice residue reached 50%, AA levels were significantly higher than in the other treatment groups. This effect may be attributed to licorice extracts modulating the intestinal microbiota by increasing beneficial bacteria, such as lactic acid bacteria and bifidobacteria, inhibiting harmful bacteria like Escherichia coli [38], and enhancing fiber fermentation [39]. Concurrently, when the addition amount of licorice residue was 25%, the concentrations of butyric acid (BA), propionic acid (PA), valeric acid (VA) and isovaleric acid (IVA) reached their highest values. This may be because sweet sorghum contains a large amount of sugars, which are used during microbial fermentation, resulting in a rapid increase in the sugar concentration. As fermentation progresses, the sugar contents are depleted, and the accumulated metabolites may inhibit the growth of microorganisms, resulting in reduced levels of these short-chain fatty acids [40]. Furthermore, Zhang et al. [41] reported that the inclusion of polysaccharide-containing substances to the feed increased the concentrations of AA and PA in the cecum, leading to improved growth performance and immune function in broilers. The test results are basically similar to the current research results.

### 4.5. Microbial Diversity

The balance of intestinal microorganisms is essential for the health of meat rabbits. Previous research showed that environmental conditions and optimized feed formulations can effectively regulate the gut microbiota, thereby improving growth performance and overall health [42]. The analysis of α-diversity in the present study showed that the diversity and richness measured by the Faith-PD and observed species indices initially decreased and then increased as the proportion of licorice residue in the feed rose. However, at a proportion of licorice residue of 100%, both indicators decreased. Similarly, the Chao and Simpson indices first decreased and then increased as the amount of licorice residue increased, indicating that excessive licorice residue reduced microbial diversity and richness. These findings are consistent with those of Ye et al. [43] and may be due to the presence of bioactive components in licorice residue, such as glycyrrhizic acid and glycyrrhetinic acid, which possess antibacterial properties [44] that inhibit the growth of certain intestinal microbes. An et al. [45] further reported that some licorice-derived components can interfere with microbial energy metabolism pathways (e.g., glycolysis or short-chain fatty acid biosynthesis), limiting microbial growth.

PCoA analysis demonstrated that varying ratios of sweet sorghum and licorice residue significantly altered the cecal microbial composition of meat rabbits, further confirming that dietary treatments effectively shaped gut community structure.

Feed nutrients such as carbohydrates, proteins, and fats are known to directly influence microbial diversity and functional capacity [46]. In the present study, the dominant cecal phyla were Firmicutes, followed by Bacteroidota and Verrucomicrobiota, which aligns with the results of previous studies [47,48]. However, the intestinal bacterial community changed significantly in response to different proportions of particle feed, resulting in the coexistence of Firmicutes and Proteobacteria. Licorice residue contains substantial amounts of non-starch polysaccharides (e.g., cellulose and hemicellulose), which serve as major fermentation substrates for cecal microbes 11. Firmicutes ferment these fibers to produce short-chain fatty acids, and these metabolites may provide carbon sources and energy that support the growth of Proteobacteria [49]. Additionally, it is possible that bioactive compounds in licorice residue inhibit other competing bacteria, potentially allowing Firmicutes and Proteobacteria to proliferate. For instance, plant extracts, such as tannins and saponins, can remarkably suppress the growth of hyperammonogenic bacteria, belonging mainly to Proteobacteria [50]. It should be noted that excess amounts of licorice residue may disrupt the balance of the flora, leading especially to excessive proliferation of Proteobacteria, which can cause various intestinal diseases.

At the genus level, Faecousia was the dominant genus, degrading carbohydrates and decomposing fibrous substrates [51]. The findings of the present study showed that the relative abundance of Faecousia peaked when the licorice residue content was 50%. In addition, Akkermansia and Faecalibaculum abundance increased significantly when 25% licorice residue was added. This may be because isoliquiritigenin (ILG) in licorice not only alters the structure of the gut microbiota but also aids in repairing the intestinal barrier [52]. Consequently, ILG in licorice residue indirectly influences the growth environment of Faecousia and Faecalibaculum by promoting the proliferation of Akkermansia, thereby enabling these bacteria to reach their peak levels in the cecum. Meanwhile, when the licorice residue was 100%, the abundance of SFMI01 and Gemmiger_A was the highest, likely due to glycyrrhizic acid and flavonoids that improve microbial proliferation by modulating intestinal pH and redox balance [53].

Further analysis showed that the five treatment groups were different in the microbial flora of taxa at various classification levels. The species levels of Lachnospirales, Lachnospiraceae, Bacilli, and SFMI01 were the most abundant in the groups containing 0%, 25%, and 100% licorice residue, and four taxa belong to the members of phylum Firmicutes. Members of Firmicutes exhibit a high diversity in the intestinal tract of animals, especially in vertebrates, with diverse species and functions. Machado et al. [54] reported that certain groups of Firmicutes across different hosts possess a unique spore formation gene spectrum, which contributes to the survival and adaptability of bacteria.

Concurrently, Dias et al. [55] found that a few members of Firmicutes are distributed across a variety of hosts and may have potential application in regulating gut microecological balance and alleviating inflammation, highlighting its key importance in the intestinal ecosystem. In addition, Bacteroidaceae and Amulumruptor were most abundant in the L50 treatment group, and both belong to the phylum Bacteroidota. Actinobacteriota had the highest abundance in the L75 group. Bacteroidota and Actinobacteriota play an essential role in the animal gut. Bacteroidota participate in the degradation of complex polysaccharides (e.g., starch and cellulose) in the intestine, thus helping the host obtain additional energy [56]. They also contribute to the production of short-chain fatty acids (such as AA and PA), which provide energy to the intestinal epithelial cells and are critical in regulating host metabolism and maintaining the integrity of the intestinal barrier [57]. It is noteworthy that, while most Bacteroidota are symbiotic bacteria, certain species, such as some Bacteroides, may become potential pathogens under specific conditions, resulting in bacterial infections. Actinobacteriota contribute to maintaining intestinal microbial balance by producing lactic acid and short-chain fatty acids, reducing intestinal pH and inhibiting the growth of potential pathogens. They also maintain intestinal mucosal barrier integrity by promoting mucin production and strengthening epithelial tight junctions [58].

## 5. Conclusions

This study demonstrated that varying the proportions of licorice residue and sweet sorghum in the diet can significantly influence the intestinal physiology and microbial community of the cecum of meat rabbits. The addition of a small amount of licorice residue to the diet of meat rabbits could improve the quality of the pellet feed as well as alter intestinal tissue morphology, VFAs, and the relative abundance of the intestinal flora in Ira meat rabbits. Based on this study’s findings, a 25% content of licorice residue (L25) in the mixed pellet feed provided the most favorable physiological state of intestinal morphology and microbial flora. In later research, the experiment should be designed in combination with the digestive performance and meat quality of meat rabbits, so as to provide more accurate feasibility analysis for meat rabbits in terms of commercial value.

## Figures and Tables

**Table 1:** Analysis of licorice residue and sweet sorghum straw (% air-dry basis).

Item	DM	CP	EE	CF	NDF	ADF	Ca	TP
Licorice residue	94.16	8.64	3.61	29.18	48.25	33.31	1.95	0.98
Sweet sorghum	92.77	8.21	3.99	27.11	42.24	29.69	0.39	0.21

Note: DM = dry matter; CP = crude protein; EE = ether extract; CF = crude fiber; NDF = neutral-detergent fiber; ADF = acid-detergent fiber; Ca = calcium; TP = total phosphorus. n = 6.

**Table 2:** Composition and analysis of experimental diets (% air-dry basis).

Ingredients	Experimental Diets				
	L0	L25	L50	L75	L100
Licorice residue	0	7.5	15	22.5	30
Sweet sorghum	30	22.5	15	7.5	0
Corn	12	12	12	12	12
CaHPO_4_	0.5	0.5	0.5	0.5	0.5
Wheat meal	17.2	17.2	17.2	17.2	17.2
Rice bran	15	16	16	17	18
Soybean meal	13	12	11	10	9
Lawn grass	9	9	10	10	10
NaCl	0.3	0.3	0.3	0.3	0.3
Premix ^1^	3	3	3	3	3
Total	100	100	100	100	100
Nutrient levels ^2^					
DE/(MJ/Kg)	11.72	11.78	11.79	11.85	11.90
CP	15.36	15.43	15.42	15.49	15.56
NDF	39.85	41.14	42.81	44.10	45.38
ADF	21.87	23.55	25.57	27.25	28.93
Ca	0.83	0.82	0.81	0.81	0.8
P	0.67	0.66	0.64	0.63	0.62
Lys	0.9	0.89	0.87	0.85	0.83

Note: L0 = 0% licorice residue + 100% sweet sorghum; L25 = 25% licorice residue + 75% sweet sorghum; L50 = 50% licorice residue + 50% sweet sorghum; L75 = 75% licorice residue + 25% sweet sorghum; L100 = 100% licorice residue + 0% sweet sorghum; the following table is the same. CP = crude protein; NDF = neutral-detergent fiber; ADF = acid-detergent fiber. ^1^ The premix provides the following per kg of diet: VA 100,000 IU, VD3 30,000 IU, VE 300 mg, VK3 20 mg, VB2 80 mg, Fe 1000 mg, Cu 200 mg, Zn 500 mg, Mu 300 mg, Se 11.3 mg, P 1.5%. ^2^ DE was a calculated value, while the others were measured values.

**Table 3:** Effect of different proportions of licorice residue and sweet sorghum on the quality of meat rabbit pelleted feed.

Items	L0	L25	L50	L75	L100	SEM	*p*-Value		
							Treatment	Linear	Quadratic
Length, cm	2.24	2.24	2.25	2.26	2.24	0.01	0.964	0.754	0.859
Hardness, N	14.35 ^ab^	13.56 ^b^	11.72 ^c^	11.89 ^c^	15.71 ^a^	0.45	0.001	0.529	<0.001
Powder content, %	0.30 ^b^	0.27 ^b^	0.47 ^a^	0.60 ^a^	0.21 ^c^	0.04	0.008	0.489	0.007
Pulverization rate, %	2.58	3.53	3.35	1.18	2.98	0.31	0.084	0.403	0.885
Briquetting ratio, %	96.43	97.20	96.58	96.22	95.69	0.22	0.289	0.12	0.2
Volume-weight, g	643.72 ^a^	561.14 ^c^	613.81 ^b^	623.70 ^b^	615.05 ^b^	7.42	<0.001	0.634	<0.001

Note: SEM = standard error of the means. n = 6. Values within the same row sharing the same or no superscripts suggest no significant difference (*p* > 0.05), while different lowercase superscripts mean significant differences (*p* < 0.05).

**Table 4:** Effects of different proportions of licorice residue and sweet sorghum straw on growth performance of meat rabbits.

Items	L0	L25	L50	L75	L100	SEM	*p*-Value		
							Treatment	Linear	Quadratic
IBW; g	723.02	721.46	720.90	724.59	722.96	7.40	0.893	0.955	0.949
FBW; g	2038.34 ^b^	2169.61 ^a^	2077.94 ^b^	2056.69 ^b^	2090.44 ^b^	11.26	0.006	0.905	0.152
ADG; g/d	31.32 ^b^	34.48 ^a^	32.29 ^b^	31.72 ^b^	31.79 ^b^	0.29	0.003	0.341	0.045
ADFI; g/d	137.41	136.47	137.73	138.28	137.19	0.52	0.865	0.717	0.824
F/G	4.40 ^a^	3.97 ^b^	4.27 ^a^	4.39 ^a^	4.37 ^a^	0.04	0.006	0.217	0.065

Note: IBW = initial body weight; FBW = final body weight; ADG = average daily gain; ADFI = average daily feed intake; F/G = ratio of feed to body weight gain; SEM = standard error of the means. n = 12. The same or no superscript letter in a row indicates no significant difference (*p* > 0.05), while different superscript letters indicate a significant difference (*p* < 0.05).

**Table 5:** Influence of licorice residue and sweet sorghum on intestinal tissue morphology in meat rabbits with different proportions.

Items, µm	L0	L25	L50	L75	L100	SEM	*p*-Value		
							Treatment	Linear	Quadratic
Duodenum									
VH	840.97 ^b^	1069.70 ^a^	706.37 ^c^	870.59 ^b^	784.64 ^b^	34.47	<0.001	0.01	0.4
VW	103.05	206.47	189.09	143.27	183.49	13.26	0.06	0.212	0.104
CD	92.18	115.84	121.63	86.34	114.9	5.48	0.134	0.641	0.444
MT	94.89 ^b^	96.55 ^b^	48.60 ^c^	114.99 ^a^	92.62 ^b^	6.07	<0.001	0.309	0.002
V/C	9.12 ^a^	9.23 ^a^	5.80 ^b^	10.08 ^a^	6.83 ^b^	0.50	0.003	0.072	0.729
Jejunum									
VH	635.56	622.05	710.83	600.91	617.93	13.93	0.076	0.492	0.172
VW	72.07 ^c^	95.04 ^b^	104.38 ^a^	85.86 ^c^	88.96 ^b^	3.36	0.008	0.131	0.003
CD	80.68 ^bc^	110.38 ^a^	97.49 ^b^	67.50 ^c^	77.00 ^bc^	4.93	0.012	0.053	0.06
MT	78.22 ^c^	120.68 ^a^	91.03 ^bc^	111.92 ^ab^	85.48 ^c^	5.54	0.038	0.847	0.029
V/C	7.94	5.72	7.33	8.93	8.32	0.380	0.050	0.085	0.221
Ileum									
VH	677.53 ^a^	693.19 ^a^	620.79 ^ab^	578.79 ^b^	556.01 ^b^	16.62	0.005	<0.001	0.591
VW	68.07 ^d^	75.29 ^d^	101.04 ^a^	84.53 ^c^	95.04 ^ab^	3.68	0.002	0.001	0.062
CD	87.39	115.38	65.15	122.14	78.94	7.91	0.078	0.826	0.524
MT	103.06	131.67	98.71	112.64	97.01	5.13	0.176	0.355	0.3
V/C	7.03 ^b^	6.14 ^bc^	9.58 ^a^	4.99 ^c^	8.54 ^ab^	0.54	0.037	0.471	0.805

Note: VH = villus height; VW = villus width; CD = crypt depth; MT = muscular thickness; V/C = villus height/crypt depth; SEM = standard error of the means. n = 12. The same or no superscript letter in a row indicates no significant difference (*p* > 0.05), while different superscript letters indicate a significant difference (*p* < 0.05).

**Table 6:** Influence of licorice residue and sweet sorghum on volatile fatty acids in cecum of meat rabbits with different levels.

Items	L0	L25	L50	L75	L100	SEM	*p*-Value		
							Treatment	Linear	Quadratic
pH	6.85	6.90	7.08	7.00	6.95	0.04	0.54	0.345	0.235
NH_3_-N	13.1	13.71	12.61	13.8	12.61	0.41	0.867	0.791	0.743
AA, %	63.39 ^c^	72.27 ^b^	75.92 ^a^	68.46 ^c^	60.75 ^d^	1.67	0.002	0.177	<0.001
BA, %	10.23	16.26	14.12	8.46	9.96	1.13	0.14	0.255	0.155
PA, %	11.23	13.03	11.37	8.31	9.77	0.77	0.41	0.186	0.752
VA, %	1.67	2.67	2.08	1.58	1.94	0.16	0.19	0.59	0.333
IVA, %	0.96	1.16	1.14	1.01	0.98	0.07	0.873	0.842	0.4
IBA, %	1.19	1.50	1.46	1.37	1.25	0.08	0.759	0.964	0.239
HA, %	0.07	0.09	0.04	0.01	0.04	0.01	0.088	0.023	0.689

Note: AA = acetic acid; BA = butyric acid; PA = propionic acid; VA = valeric acid; IVA = isovaleric acid; IBA = isobutyric acid; HA = hexanoic acid; pH = potential of hydrogen. SEM = standard error of the means. n = 12. The same or no superscript letter in a row indicates no significant difference (*p* > 0.05), while different superscript letters indicate a significant difference (*p* < 0.05).

## Data Availability

The raw sequence data reported in this paper have been deposited in the Genome Sequence Archive (Genomics, Proteomics & Bioinformatics 2025) in the National Genomics Data Center (Nucleic Acids Res 2025), China National Center for Bioinformation/Beijing Institute of Genomics, Chinese Academy of Sciences (GSA: CRA036065) that are publicly accessible at https://ngdc.cncb.ac.cn/gsa (accessed on 25 December 2025).

## Supplementary Materials

The following supporting information can be downloaded at: https://www.mdpi.com/article/10.3390/microorganisms14040868/s1, Table S1: Analysis of α diversity of intestinal microflora; Table S2: The relative abundance of intestinal flora in meat rabbits at the phylum level (%); Table S3: The relative abundance of intestinal flora in meat rabbits at the genus level (%).
