# Supplementary material for: Effects of Different Levels of Licorice Residue and Sweet Sorghum on Pellet Feed Quality, Intestinal Morphology, Cecal Volatile Fatty Acids, and Microorganisms in Meat Rabbits
Source: Microorganisms. 2026 Apr 12;14(4):868. doi: 10.3390/microorganisms14040868 (PMC13119021; doi:10.3390/microorganisms14040868)
Supplement: Supplementary file 1 [file microorganisms-14-00868-s001.zip › supplementary materials2/supplementary materials.docx]

**Table S1** Analysis of α diversity of intestinal microflora

| **Item** | **L0** | **L25** | **L50** | **L75** | **L100** | **SEM** | ***P*-value** | | |
| --- | --- | --- | --- | --- | --- | --- | --- | --- | --- |
|  |  |  |  |  |  |  | **Treatment** | **Linear** | **Quadratic** |
| Chao1 | 1580.22 | 1422.29 | 1417.53 | 1501.72 | 1487.62 | 26.91 | 0.31 | 0.58 | 0.10 |
| Faith_pd | 91.47^a^ | 80.31^b^ | 82.34^b^ | 88.78^ab^ | 81.58^b^ | 1.40 | 0.03 | 0.20 | 0.24 |
| Goods_coverage | 1.00 | 1.00 | 1.00 | 1.00 | 1.00 | ＜0.01 | 0.33 | 0.71 | 0.08 |
| Observed_species | 1545.45 | 1386.68 | 1385.75 | 1472.67 | 1439.57 | 25.90 | 0.26 | 0.49 | 0.12 |
| Pielou_e | 0.86^a^ | 0.84^b^ | 0.85^ab^ | 0.86^a^ | 0.86^a^ | ＜0.01 | 0.02 | 0.37 | 0.14 |
| Shannon | 9.11 | 8.77 | 8.88 | 9.08 | 8.98 | 0.04 | 0.08 | 0.89 | 0.11 |
| Simpson | 1^a^ | 0.99^b^ | 0.99^b^ | 1^a^ | 1^a^ | ＜0.01 | 0.03 | 0.61 | 0.05 |

In the first behavior group of the table, the first column corresponds to the calculation results of the alpha diversity index of each sample at the corresponding sequencing depth. Data are expressed as means ± SEM ( n = 6 ). The same or no superscript letter in a row indicates no significant difference (*P*>0.05), while different superscript letters indicate a significant difference (*P*<0.05).

**Table S2** The relative abundance of intestinal flora in meat rabbits at the phylum level (%)

| **Item** | **L0** | **L25** | **L50** | **L75** | **L00** | **SEM** | ***P*-value** | | |
| --- | --- | --- | --- | --- | --- | --- | --- | --- | --- |
|  |  |  |  |  |  |  | **Treatment** | **Linear** | **Quadratic** |
| Firmicutes_A | 57.90^a^ | 48.74^c^ | 55.49^ab^ | 51.09^b^ | 50.32^b^ | 1.080 | 0.026 | 0.066 | 0.480 |
| Bacteroidota | 14.72 | 10.75 | 10.57 | 10.43 | 9.68 | 0.627 | 0.075 | 0.017 | 0.190 |
| Verrucomicrobiota | 3.55^b^ | 6.60^a^ | 4.28^b^ | 5.26^ab^ | 3.72^b^ | 0.342 | 0.016 | 0.634 | 0.023 |
| Firmicutes_D | 3.84^b^ | 4.80^a^ | 3.21^b^ | 3.44^b^ | 3.16^b^ | 0.161 | 0.002 | 0.005 | 0.522 |
| Actinobacteriota | 1.95^c^ | 2.59^b^ | 3.12^ab^ | 3.71^a^ | 3.12^ab^ | 0.156 | 0.001 | ＜0.001 | 0.023 |
| Desulfobacterota_I | 1.05 | 1.03 | 1.35 | 1.28 | 1.22 | 0.065 | 0.458 | 0.210 | 0.385 |
| Proteobacteria | 0.44^bc^ | 0.51^ab^ | 0.60^a^ | 0.41^c^ | 0.58^a^ | 0.018 | ＜0.001 | 0.056 | 0.490 |
| Firmicutes_C | 0.83^a^ | 0.19^b^ | 0.49^ab^ | 0.19^b^ | 0.24^b^ | 0.078 | 0.025 | 0.021 | 0.173 |
| Firmicutes_B | 0.14^ab^ | 0.17^a^ | 0.17^a^ | 0.16^a^ | 0.06^b^ | 0.013 | 0.035 | 0.072 | 0.009 |
| Patescibacteria | 0.33^a^ | 0.09^b^ | 0.06^b^ | 0.09^b^ | 0.12^b^ | 0.031 | 0.025 | 0.034 | 0.013 |
| Synergistota | 0.02^b^ | 0.01^b^ | 0.33^a^ | 0.00^b^ | 0.03^b^ | 0.031 | ＜0.001 | 1.000 | 0.002 |
| Cyanobacteria | 0.02 | 0.05 | 0.03 | 0.02 | 0.02 | 0.004 | 0.230 | 0.221 | 0.157 |
| Others | 15.21^c^ | 24.46^ab^ | 20.29^b^ | 23.89^ab^ | 27.72^a^ | 1.157 | 0.003 | 0.001 | 0.688 |

The first behavior group in the table, the first column species name. Data are expressed as means ± SEM ( n = 6 ). The same or no superscript letter in a row indicates no significant difference (*P*>0.05), while different superscript letters indicate a significant difference (*P*<0.05).

**Table S3** The relative abundance of intestinal flora in meat rabbits at the genus level (%)

| **Item** | **L0** | **L25** | **L50** | **L75** | **L00** | **SEM** | ***P*-value** | | |
| --- | --- | --- | --- | --- | --- | --- | --- | --- | --- |
|  |  |  |  |  |  |  | **Treatment** | **Linear** | **Quadratic** |
| Faecousia | 8.67 | 9.49 | 11.13 | 8.78 | 9.75 | 0.47 | 0.484 | 0.666 | 0.354 |
| SFMI01 | 6.27^ab^ | 6.42^ab^ | 7.26^a^ | 4.86^b^ | 8.04^a^ | 0.33 | 0.02 | 0.332 | 0.244 |
| Akkermansia | 3.55^b^ | 6.60^a^ | 4.27^b^ | 5.26^ab^ | 3.72^b^ | 0.34 | 0.016 | 0.632 | 0.024 |
| CAG-485 | 4.92 | 2.64 | 1.75 | 2.54 | 3.40 | 0.38 | 0.082 | 0.218 | 0.012 |
| Borkfalkia | 3.49^a^ | 2.03^b^ | 2.70^ab^ | 2.61^ab^ | 2.37^b^ | 0.15 | 0.027 | 0.092 | 0.147 |
| Gemmiger_A | 1.36^b^ | 1.68^b^ | 1.55^b^ | 1.93^b^ | 3.01^a^ | 0.18 | 0.02 | 0.003 | 0.131 |
| CAG-273 | 2.30^a^ | 1.61^b^ | 1.85^ab^ | 1.75^b^ | 1.10^c^ | 0.10 | 0.001 | ＜0.001 | 0.683 |
| UBA3282 | 2.27^a^ | 0.91^b^ | 1.41^ab^ | 2.38^a^ | 1.23^b^ | 0.18 | 0.015 | 0.575 | 0.482 |
| Acetobacterium | 1.46 | 1.57 | 1.60 | 1.84 | 1.56 | 0.10 | 0.829 | 0.536 | 0.507 |
| Onthenecus | 0.99 | 2.14 | 1.37 | 1.30 | 1.90 | 0.15 | 0.067 | 0.306 | 0.714 |
| Faecalibaculum | 1.09^b^ | 2.15^a^ | 1.16^b^ | 1.46^ab^ | 1.6^ab^ | 0.12 | 0.029 | 0.663 | 0.537 |
| CAG-269 | 0.92^b^ | 1.65^a^ | 1.61^a^ | 1.57^a^ | 0.86^b^ | 0.10 | 0.002 | 0.708 | ＜0.001 |
| UMGS1994 | 1.8^a^ | 0.76^b^ | 1.73^a^ | 1.05^ab^ | 0.68^b^ | 0.15 | 0.031 | 0.049 | 0.783 |
| Ruminococcus_D | 1.75^a^ | 0.69^b^ | 1.08^ab^ | 1.61^a^ | 0.74^b^ | 0.12 | 0.005 | 0.128 | 0.542 |
| Desulfovibrio_R | 0.92 | 0.83 | 1.17 | 1.07 | 1.11 | 0.06 | 0.382 | 0.161 | 0.717 |
| Others | 58.24 | 58.81 | 58.34 | 59.98 | 58.94 | 0.54 | 0.874 | 0.527 | 0.815 |

The first behavior group in the table, the first column species name. Data are expressed as means ± SEM ( n = 6 ). The same or no superscript letter in a row indicates no significant difference (*P*>0.05), while different superscript letters indicate a significant difference (*P*<0.05).
